# Supplementary figures and images for: LMO2 is essential to maintain the ability of progenitors to differentiate into T-cell lineage in mice
Source: eLife. 2021 Aug 12;10:e68227. doi: 10.7554/eLife.68227 (PMC8360648; doi:10.7554/eLife.68227)

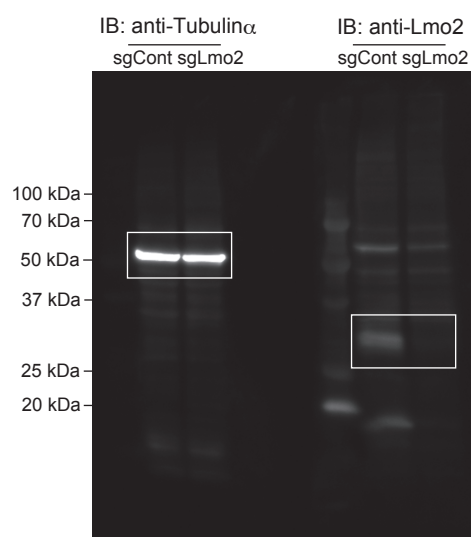

Supplement: Figure 2—figure supplement 1—source data 1. [file elife-68227-fig2-figsupp1-data1.pdf]
